# Heme A Synthase Deficiency Affects the Ability of *Bacillus cereus* to Adapt to a Nutrient-Limited Environment

**DOI:** 10.3390/ijms23031033

**Published:** 2022-01-18

**Authors:** Alice Chateau, Béatrice Alpha-Bazin, Jean Armengaud, Catherine Duport

**Affiliations:** 1 Biology Department, Campus Jean-Henri Fabre, Avignon Université, INRAE, UMR SQPOV, 84914 Avignon, France; alice.chateau-huyot@univ-avignon.fr; 2 Département Médicaments et Technologies pour la Santé (DMTS), Université Paris Saclay, CEA, INRAE, SPI, 30200 Bagnols-sur-Cèze, France; beatrice.alpha-bazin@cea.fr (B.A.-B.); jean.armengaud@cea.fr (J.A.)

**Keywords:** *Bacillus cereus*, heme A synthase, aerobic respiration, proteome

## Abstract

The branched aerobic respiratory chain in *Bacillus cereus* comprises three terminal oxidases: cytochromes *aa3*, *caa3*, and *bd*. Cytochrome *caa3* requires heme A for activity, which is produced from heme O by heme A synthase (CtaA). In this study, we deleted the *ctaA* gene in *B. cereus* AH187 strain, this deletion resulted in loss of cytochrome *caa3* activity. Proteomics data indicated that *B. cereus* grown in glucose-containing medium compensates for the loss of cytochrome *caa3* activity by remodeling its respiratory metabolism. This remodeling involves up-regulation of cytochrome *aa3* and several proteins involved in redox stress response—to circumvent sub-optimal respiratory metabolism. *CtaA* deletion changed the surface-composition of *B. cereus,* affecting its motility, autoaggregation phenotype, and the kinetics of biofilm formation. Strikingly, proteome remodeling made the *ctaA* mutant more resistant to cold and exogenous oxidative stresses compared to its parent strain. Consequently, we hypothesized that *ctaA* inactivation could improve *B. cereus* fitness in a nutrient-limited environment.

## 1. Introduction

*Bacillus cereus* is a ubiquitous endospore-forming bacterium, which mainly affects humans as a food-borne pathogen [1]. Recently, we examined the ability of the emetic strain *B. cereus* AH187 (F4810/72) to survive oligotrophic conditions encountered in groundwater. Our results showed that vegetative *B. cereus* cells rapidly evolved to produce a mixed population composed of endospores and asporogenic variants bearing mutations in the *spo0A* gene, which encodes a master regulator for entry into sporulation [2]. The whole genome of one of the variants isolated was sequenced, and the mutations identified included an alteration to codon 178 of *ctaA* gene (GCT→ACT, Ala→Thr). This variant survives in sterilized groundwater over a long period in a vegetative form and has a competitive advantage compared to its parental strain [2].

**Figure 1:**
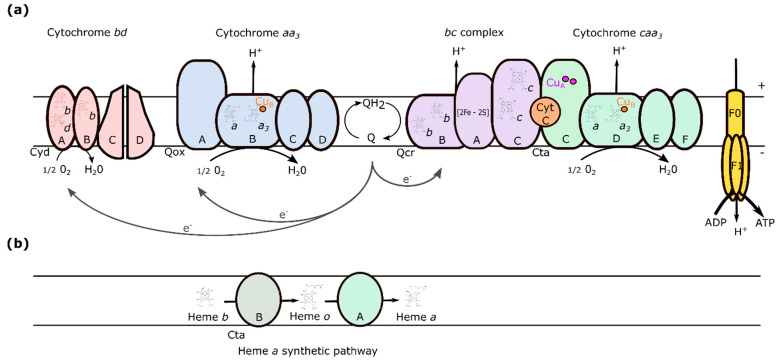
The branched aerobic respiratory chain in *Bacillus cereus*. (**a**) Schematic representation of the electron transport chain in the cytoplasmic membrane. Menaquinone (Q) is reduced to menaquinol (QH_2_), electrons (e^−^) are transferred to cytochrome *caa3*, *aa3*, and *cyd* terminal oxidases to reduce oxygen to H_2_O. The resulting electrochemical gradient is used by ATP synthase to produce ATP. QH_2_ provides electrons to cytochrome *caa3* (green) via cytochrome *bc* (purple) and cytochrome *c* (orange). Alternatively, electrons from QH_2_ can be delivered directly to cytochrome *aa3* (blue) or cytochrome *bd* (pink) menaquinol oxidases, the latter is not a proton pump. Cytochromes *caa3* and *aa3* have four subunits encoded by the *ctaCDEF* and *qoxABCD* operons, respectively. Subunit I of both enzymes carries the electron-accepting heme *a* that delivers electrons to the active site—composed of heme *a3* and a copper center (Cu_B_). (**b**) Heme *a* synthesis pathway. Heme *a* synthesis is catalyzed by CtaB and CtaA—heme *o* and *a* synthases, respectively.

The *ctaA* gene encodes heme A synthase (CtaA), a membrane-bound enzyme that converts heme O to heme A. CtaA is required for cytochrome *caa3* oxidase biosynthesis and sporulation in *Bacillus subtilis* [3]. *B. cereus* cytochrome *caa3* is made up of four proteins (CtaCDEF), with Cu_A_ and a cytochrome *c* domain in subunit II (CtaC). This protein may form a supercomplex with the cytochrome *bc* complex (QcrABC) and cytochrome *c*_550_ (CccA) or cytochrome *c*_551_ [4] in the membrane, as reported in *B. subtilis* [5]. Cytochrome *caa3* is one of the two heme-copper terminal oxidases in the branched *B. cereus* aerobic respiratory chain [6,7] (Figure 1). The other is cytochrome *aa3*, which uses menaquinol as electron donor instead of cytochrome *c*. In contrast to cytochrome *caa3*, cytochrome *aa3* is not strictly dependent on heme A for its activity, as it can also use heme B and heme O to produce a novel *bo_3_* cytochrome [8]. The third terminal oxidase in the *B. cereus* respiratory chain is a cytochrome *bd* menaquinol oxidase that requires neither copper nor heme A for activity. Like cytochrome *caa3*, cytochrome *aa3*, and cytochrome *bd* are also four-protein complexes, composed of QoxABCD and CydABCD, respectively [9].

The composition of the respiratory chain is regulated by growth conditions [10]. Thus, the cytochrome *aa3*-terminating branch of the *B. subtilis* respiratory chain is the major contributor to respiration in most aerobic growth conditions, whereas the cytochrome *caa3*-terminating branch is a minor contributor [11]. The branch terminating at the *bd*-oxidase was shown to contribute to microaerobic respiration in *B. subtilis* [12]. Respiratory flexibility is an important factor that allows bacteria to cope with changing oxygen and nutrient conditions.

Here, we investigated the impact of functional loss of *ctaA* on *B. cereus*, first discovered in an environment with limited nutrients. Proteomics analysis revealed that the mutant strain adapts its respiratory network. Thus, lack of CtaA disrupted electron flow through the *bc*-*caa3* pathway, and upregulated the menaquinol-cytochrome *aa3* oxidase pathway. Re-routing of respiratory chain electron transport causes endogenous redox stress and is accompanied by changes at the bacterial surface.

## 2. Results

### 2.1. B. cereus AH187 CtaA Is Required for Cytochrome Aa3 Oxidase Activity, and Optimal Growth

**Figure 2:**
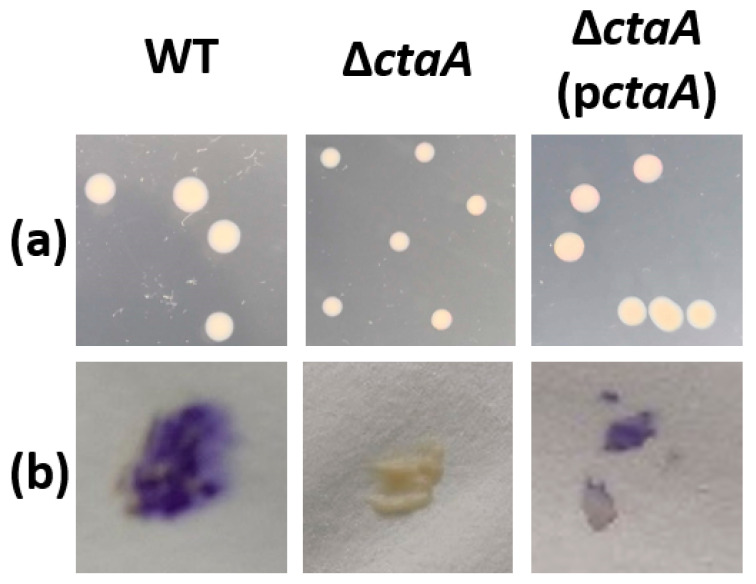
Phenotypes of *B. cereus* AH187 WT, ∆*ctaA* and *ctaA*-complemented Δ*ctaA* (p*ctaA*) colonies. (**a**) Colony morphologies on LB agar plates following 18 h of incubation at 30 °C. All images are shown at the same magnification. (**b**) Oxidase activity as measured by TMPD colorimetric assay. Oxidase-positive colonies are stained purple.

To investigate the role of CtaA in wild-type *B. cereus* AH187 (WT), the *ctaA* gene (BCAH187_A4064) was deleted. On Lysogeny Broth (LB) agar plates, Δ*ctaA* strain colonies were smaller than those of its parent strain, indicating a growth defect on solid medium (Figure 2a). Colonies were tested for N, N, N′, N′-tetramethyl-p-phenylenediamine (TMPD) oxidation capacity. This artificial electron donor interacts specifically with cytochrome *caa3* oxidase [13], producing blue staining of enzymatically-active colonies. Oxidation activity was detectable in WT and complemented strains, but not in the ∆*ctaA* strain (Figure 2b), confirming a lack of cytochrome *caa3* activity in these colonies.

**Figure 3:**
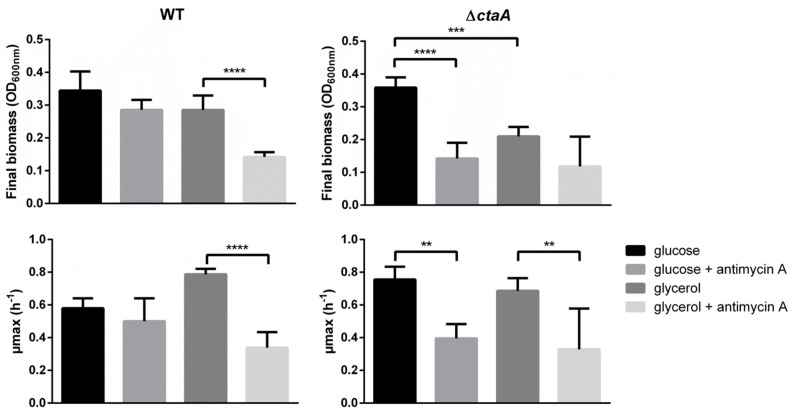
Growth parameters (µ_max_ and final biomass) of *B. cereus* AH187 WT and ∆*ctaA* strains in MOD medium supplemented with 30 mM glucose or 60 mM glycerol, in the presence or absence of antimycin A. Cultures were performed in microplates. Statistical analysis was performed by one-way ANOVA and Tukey’s *post hoc* analysis. **: *p* < 0.01, ***: *p* < 0.001, ****: *p* < 0.0001.

In liquid MOD medium supplemented with 30 mM glucose (MODG) [14], the ∆*ctaA* mutant grew at the same rate and reached the same final biomass as its parent strain (Figure S1). However, it excreted more acetate (yield 1.11 ± 0.15 mol/mol glucose) than its parent strain (yield 0.48 ± 0.06 mol/mol glucose), indicating increased overflow metabolism. When glucose was replaced by glycerol, the ∆*ctaA* mutant showed altered growth (Figure 3), suggesting that lack of CtaA and *caa3* activity reduced the capacity of *B. cereus* to use non-phosphotransferase-system-dependent glycerol as a carbon source [15]. To confirm the role of *caa3* activity in carbon metabolism, antimycin A—which selectively binds to the *bc* complex and interrupts cytochrome *caa3*’s function [16]—was added to growth medium. As expected in these conditions [6], growth of WT strain was altered on glycerol but not on glucose. Unexpectedly, antimycin A impaired ∆*ctaA* mutant growth on both glucose and glycerol (Figure 3), suggesting that in the absence of CtaA, this compound inhibits membrane-centered transport processes [17].

### 2.2. Proteome and Exoproteome Response to CtaA Deficiency

To determine how CtaA deficiency affects cellular metabolism at distinct growth phases on MODG medium, we performed shotgun proteomics assays on three biological replicates for Δ*ctaA* and WT strains at three time-points. The time-points were: EE (early exponential growth phase, OD = 0.1), LE (late exponential growth phase, OD = 1), and S (stationary growth phase, OD = 1.5) (Figure S1). The proteomics dataset acquired on the 36 samples (2 strains × 3 time-points × 3 replicates for cellular proteome and exoproteome) comprised 843,332 MS/MS spectra and a total of 27,804 validated peptide sequences. From these results, based on the confident detection of at least two distinct peptides per protein, 1922 proteins were identified in the cellular proteome (Table S1), and 998 proteins in the exoproteome (Table S2).

#### 2.2.1. Cellular Proteome

**Figure 4:**
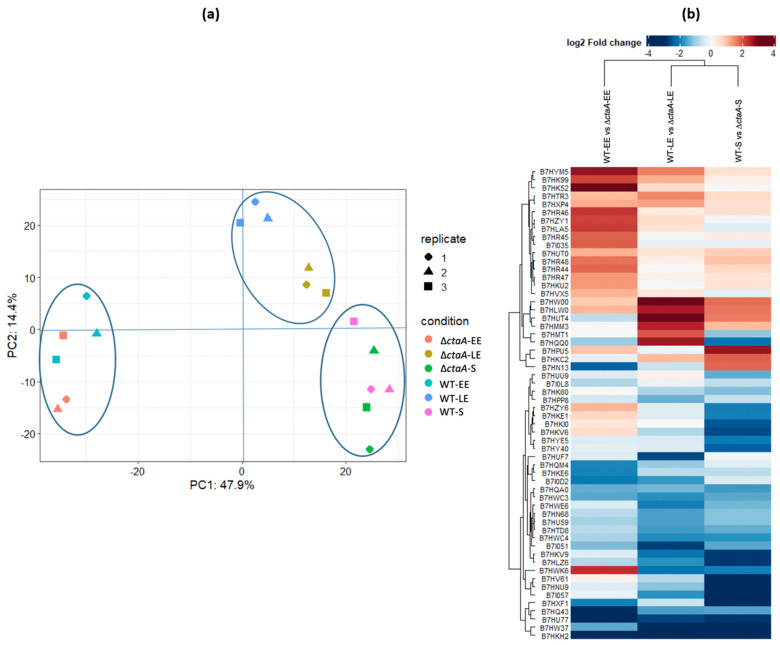
Cellular proteome remodeling in Δ*ctaA* mutant compared to its parental *B. cereus* AH187 strain (WT) at early exponential (EE), late exponential (LE), and stationary (S) growth phases. (**a**) Principal component analysis (PCA) showing reproducibility of WT and Δ*ctaA* biological replicates and the dynamics of WT and Δ*ctaA* cellular proteomes described by the first two components, PC1 and PC2. PC1 and PC2 explained 47.9% and 14.4% of total data variability, respectively. Replicates in each condition were plotted as a function of their PC1 and PC2 values. (**b**) Heat map of the 58 differentially-accumulated proteins (DAPs) showing their abundance changes (log_2_ fold-change) in each growth phase. DAPs are indicated by their UniProt ID. Ascending hierarchical classification was determined based on Euclidean distance. The color code is as follows: red for down-DAPs, and blue for up-DAPS.

Principal component analyses (PCA) revealed good homogeneity of the replicates for each growth phase (Figure 4a). PCA also indicated that EE samples clearly segregated from LE and S samples, and that Δ*ctaA* and WT samples showed poor convergence at LE compared to EE and S growth phases. These results indicate that the cellular proteome undergoes relatively few changes to support *B. cereus* growth in the absence of CtaA. Pairwise comparisons identified differentially accumulated proteins (DAPs) between Δ*ctaA* and WT strains at each growth phase. Only proteins with an adjusted *p*-value ≤ 0.05 and at least a 1.5-fold-change (|log_2_ fold-change| ≥ 0.56) were considered to be differentially accumulated between the two strains. Overall, 58 DAPs were identified with high confidence, with 24 proteins less abundant (down-DAPs), and 34 more abundant (up-DAPs) in Δ*ctaA* compared to WT (Table S3). The distribution of these proteins at the three growth phases is illustrated in Figure 4b. Among the 24 down-DAPs, whatever the growth phase, FliC flagellin (B7HLW0)—a major component of the surface-associated flagellum [18]—was significantly less abundant in Δ*ctaA* than in the WT strain (adjusted *p*-value < 10^−4^). Flagellin-based motility may thus be compromised in the absence of CtaA. Another surface-associated protein, B7HXP4, which is potentially one of the two components of the *B. cereus* S-layer [2], was also less abundant in Δ*ctaA* than in WT strains at both EE and S growth phases, suggesting altered surface integrity. The down-DAP B7HLA5, identified at EE growth phase, shares homologies with the *B. subtilis* RicA protein. RicA is a component of the RicAFT complex that senses the cellular redox status, and accelerates phosphorylation of the Spo0A transcriptional regulator [19]. Decreased abundance of RicA in the absence of CtaA could affect the ability of *B. cereus* to form biofilm and/or to sporulate [20,21]. Eight other down-DAPs identified at EE growth phase are linked to the iron acquisition system (Table S3). The proteins B7HR44 (DhbA/BacA), B7HR45 (DhbB/BacC), B7HR46 (DhbC/BacE), B7HR47 (DhbE/BacB) and B7HR48 (DhbF/BacF) are involved in synthesizing the iron-binding bacillibactin siderophore [22,23]. Related proteins were also down-regulated, including FeuA (B7HKU2), a siderophore-binding protein [24], and IlsA (B7HK52), a surface protein which plays an important role in iron acquisition in *B. cereus* [25]. In addition, Dps2 (B7HVX5), an iron-binding protein playing a role in iron storage and resistance to oxidative stress [26] was also among the down-DAPs. The significant decrease in abundance of these eight proteins in the Δ*ctaA* strain suggests down-regulation of siderophore-mediated iron uptake, potentially preventing accumulation of an excess of free intracellular iron which could lead to ROS generation via the Fenton reaction [27]. Interestingly, we noted the presence of a nitroreductase-like protein (B7HMT1) among the down-DAPs. Low amounts of nitroreductase could prevent the accumulation of its reduced substrate: quinones [28], which have also been reported to enhance the Fenton reaction [29].

Among the 34 up-DAPS, three proteins were significantly more abundant in the Δ*ctaA* compared to the WT strain whatever the growth phase (Table S3). These were the UCPA oxidoreductase B7HLZ6 of unknown function, the disulfide bond-formation protein D precursor (BdbD, B7HU77), and the flavohemoglobin Hmp (B7HKH2). BdbD is involved in disulfide bond-formation in extra-cytoplasmic proteins, and contributes to various cellular processes, including maturation of cytochrome *c* and endospore maturation [30,31]. Hmp is known to protect bacteria from NO/redox stress [32], and is activated by the two-component ResDE system in response to reduced menaquinone accumulation [33]. ResDE also activates the cytochrome and heme biogenesis pathways. Accordingly, among up-DAPs, we found the first two subunits of quinol oxidase *caa3*–QoxB (B7HWC4) and QoxA (B7HWC3) (Figure 4)—alongside HemC (B7HQM4), which is involved in the heme sub-pathway that synthesizes coproporphyrinogen-III from 5-aminolevulinate. The other up-DAPs included: (i) two sulfatases (B7HKE1 and B7HKV9) and one sulfate adenyltransferase Sat (B7HKE6) that regulate and contribute to the sulfate assimilation pathway [34], which is known to be upregulated in response to oxidative stress [35]. (ii) An enzyme involved in molybdopterin co-factor (MoCo) biosynthesis (MoeB, B7HW37) that contributes to maintaining intracellular sulfur and thiol homeostasis [36] and prevents ROS damage [37]. (iii) a KefF quinone oxidoreductase-like protein (B7HUU9), that could decrease the redox toxicity of quinones and activate the potassium efflux system [38]. (iv) Eight sporulation-associated proteins comprising four spore components (B7I0D2, B7HXF1, B7HU59, B7I057) and four regulators of stages in the sporulation process—Spo0M (B7I0D2, stage 0), SpoII0Q (B7HY40, stage II), and RfsA and SpoIIIAN (B7HYE5 and B7HNU9, stage III). Up-regulation of these sporulation-associated proteins during exponential growth of the Δ*ctaA* mutant suggests that a signal that normally makes sporulation a post-exponential growth-phase response in WT strains could be detected earlier in Δ*ctaA*. Our proteomics data suggest that this signal could be redox stress, which Δ*ctaA* cells are exposed to from the beginning of growth.

#### 2.2.2. Exoproteome Analysis

**Figure 5:**
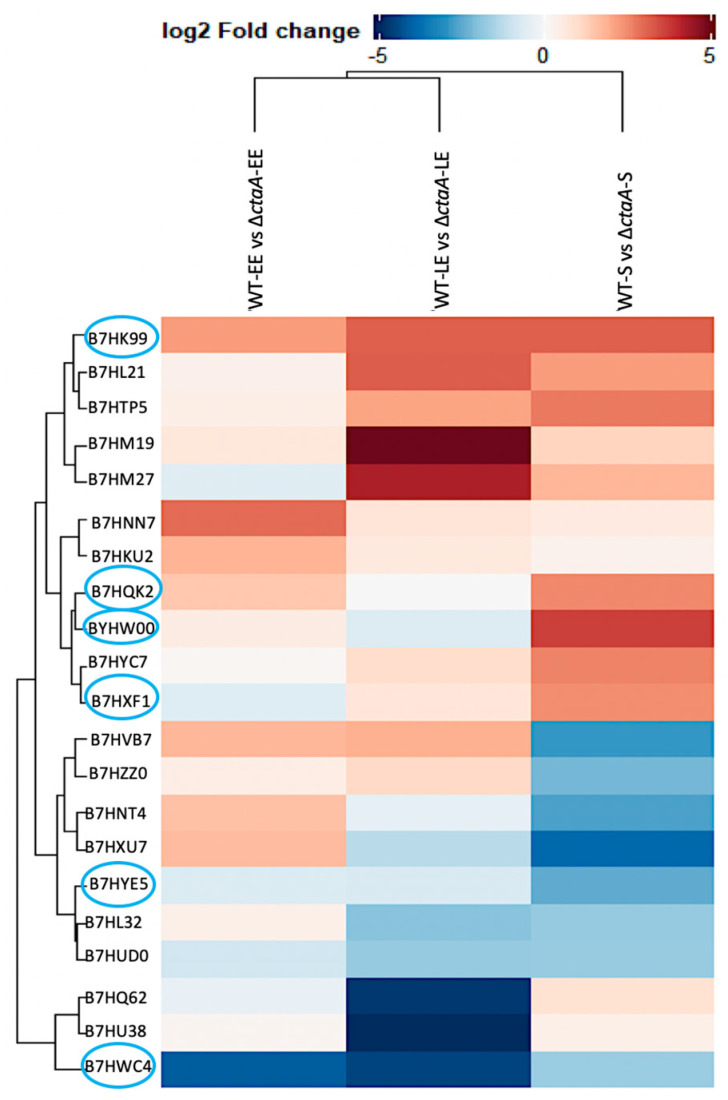
Heat map showing the growth phase distribution of the 21 differentially accumulated proteins (DAPs) identified in the Δ*ctaA* exoproteome compared to the WT exoproteome. DAPs are indicated by their UniProt ID, and log_2_ fold-change values are given for early exponential (EE), late exponential (LE), and stationary (S) growth phases. Circled DAPs correspond to those identified both in the cellular proteome and the exoproteome. Ascending hierarchical classification was determined based on Euclidean distance. The color code is as follows: red for down-DAPs, and blue for up-DAPS.

According to our quantitative and statistical criteria, only 21 exoproteins differentially accumulated between the strains (Table S4). These DAPs were mainly related to intracellular processes, and consequently are not classical secreted proteins [39]. However, six of them were identified both in the exoproteome and in the cellular proteome, including the membrane-associated iron ABC transporter (FeuA, B7HKU2) and the transmembrane quinol oxidase subunit 2 (QoxA, B7HWC4) (Figure 5). These corroborating results confirm their abundance-changes in Δ*ctaA* compared to WT strain.

### 2.3. Phenotypic Characterization of CtaA-Deficient B. cereus AH187 Strain

#### 2.3.1. Resistance to Stress

**Figure 6:**
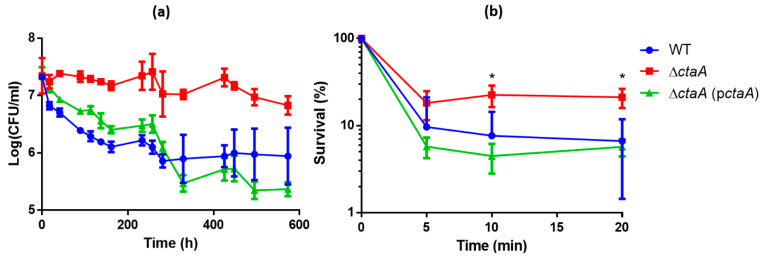
Response to cold (**a**) and oxidative (**b**) stresses by *B. cereus* AH187 WT, ∆*ctaA* and *ctaA*-complemented ∆*ctaA* strains. (**a**) *B. cereus* WT (blue), ∆*ctaA* (red) and complemented ∆*ctaA*(p*ctaA*) (green) strains were incubated in MODG at 4 °C. Numbers of colony forming units (CFU) were monitored over time. Values correspond to mean ± SD measured for three biological replicates. Wilcoxon test, *p* < 0.0001 for WT vs. ∆*ctaA* and complemented ∆*ctaA* vs. ∆*ctaA*. (**b**) *B. cereus* AH187 WT (blue), ∆*ctaA* (red) and complemented ∆*ctaA*(p*ctaA*) (green) cells were grown in MODG at 30 °C to the mid-exponential growth phase, before exposure to 10 mM H_2_O_2_ for 5, 10, or 20 min. Surviving CFU were counted and expressed as (N/N0) × 100. Values correspond to mean ± SD measured for three biological replicates. Statistical analysis was performed by one-way ANOVA followed by Tukey’s post hoc analysis. *: *p* < 0.05.

Proteomics data suggested that in the absence of CtaA, having already activated their stress-response, cells should better resist additional exogenous stress. We assessed the ability of WT, ∆*ctaA* and complemented strains to survive at temperatures below the minimal growth temperature (i.e., below 8 °C [40]), and to resist exogenous oxidant. Viable colony forming units (CFU) counts decreased regularly over time during incubation at 4 °C for all strains, but the viability loss was significantly greater for WT and complemented strains than for the ∆*ctaA* strain (Figure 6a). Similarly, aerobically-grown Δ*ctaA* was more resistant to the deleterious effects of H_2_O_2_ than either WT or complemented strains (Figure 6b). Taken together, these results suggest that CtaA deficiency effectively makes *B. cereus* more resistant to cold and oxidative stressors.

#### 2.3.2. Motility

**Figure 7:**
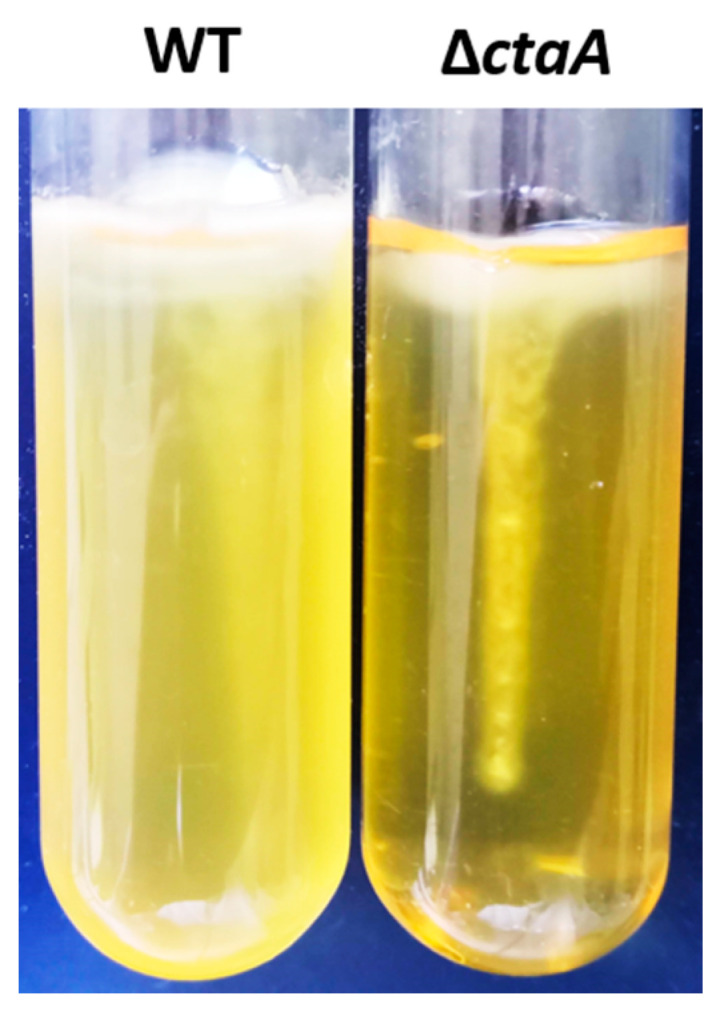
Motility of ∆*ctaA* mutant and its parental *B. cereus* AH187 strain in semisolid medium.

Proteomics data relating to altered expression of flagellum-related proteins suggested that motility could be modified in the ∆*ctaA* strain. When inoculated in semisolid medium, the motile parental strain produced diffuse turbidity as it grew. In contrast, the Δ*ctaA* mutant grew only along the line of inoculation (Figure 7), indicating that CtaA is required for *B. cereus* motility.

#### 2.3.3. Surface Properties

Proteomics analysis revealed several modifications to the abundance of proteins involved in cell wall, membrane and envelope biogenesis in the ∆*ctaA* mutant compared to its parental strain, suggesting surface alterations in this mutant.

In particular, a putative component of the S-layer (B7HXP4) was expressed at lower levels in the ∆*ctaA* mutant compared to the WT strain. To confirm these findings, we extracted non-covalently attached proteins (S-layer fraction) from the surface of bacteria for western blot analysis. B7HXP4 was confirmed to be less abundant in the S-layer fraction from the ∆*ctaA* strain compared to WT and complemented strains (Figure S2).

**Figure 8:**
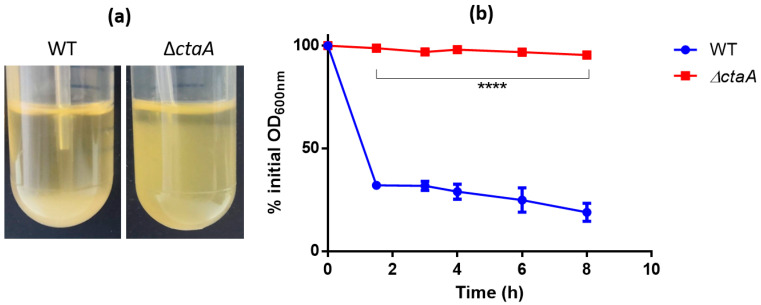
Autoaggregation of ∆*ctaA* mutant and its parental *B. cereus* AH187 strain. (**a**) Macroscopic autoaggregation assays. Autoaggregation was measured in stationary tubes after culture for 8 h in BHI medium. (**b**) Sedimentation assays. Overnight cultures in BHI medium were adjusted to an OD_600_ of 1, and then incubated under static conditions in a spectrometry cuvette at room temperature. Absorbance (OD_600_) was monitored for 8 h. Data are expressed as percentages of initial OD_600_. Experiments were performed in triplicate. Statistical significance of the observed differences was determined by two-way ANOVA with Bonferroni post hoc test. ****: *p* < 0.0001.

As autoaggregation is related to cell-surface characteristics [41], we assessed the capacity of ∆*ctaA* and WT cells to autoaggregate by performing sedimentation assays. The OD_600_ of bacterial culture suspensions was monitored during static incubation [41]. Most WT cells settled to the bottom of the tube over the course of incubation for 8 h, whereas Δ*ctaA* mutant cultures remained turbid (Figure 8a). Sedimentation kinetics revealed that WT cells aggregated rapidly, reaching 81.1 ± 4.4% autoaggregation after 8 h, whereas Δ*ctaA* have lost the autoaggregation phenotype (Figure 8b).

#### 2.3.4. Adhesion/Biofilm

**Figure 9:**
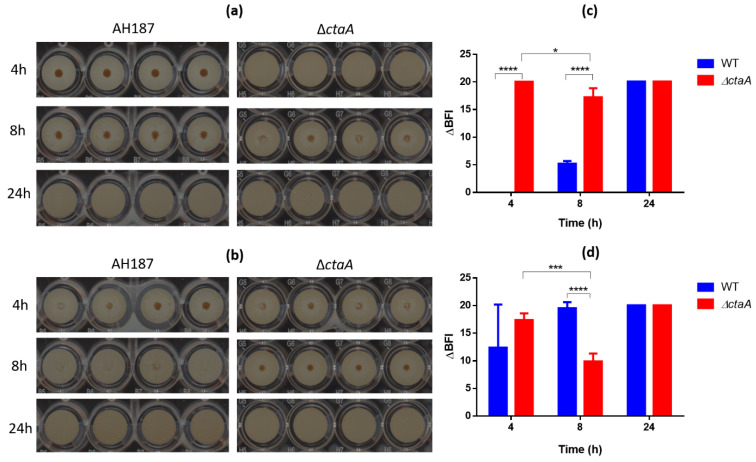
Biofilm formation kinetics of ∆*ctaA* mutant and its parental strain *B. cereus* AH187 using the Biofilm Ring Test^®^ assay. (**a**,**b**) Biofilm profiling of ∆*ctaA* and WT strains according to their ability to immobilize magnetic microbeads after 4, 8 and 24 h of incubation at 25 °C (**a**) and 30 °C (**b**). Images were obtained after magnetization of the plates on the block test and scanning with the plate reader. (**c**,**d**) Analysis of microplate images by the BioFilm Control Elements software at 25 °C (**c**) and 30 °C (**d**). Error bars correspond to the standard deviation of the mean of three replicates. Unpaired student *t*-test. *: *p* < 0.05, ***: *p* < 0.001, ****: *p* < 0.0001.

As both motility and autoaggregation can contribute to biofilm formation [42], we used the BioFilm Ring Test^®^ [43] to assess whether the Δ*ctaA* mutant could attach to a solid surface and develop a sessile biomass. Both Δ*ctaA* and WT strains formed biofilm on microplates after 24 h of incubation. However, the kinetics of biofilm formation differed between the two strains (Figure 9). Thus, although after 4 h at 25 °C and 30 °C ∆*ctaA* cells had formed a biofilm (ΔBFI > 17, Figure 9c,d), this biofilm did not persist over time, as revealed by the decrease in ΔBFI at 8 h. In contrast, this value increased progressively for WT cells from 8 h incubation, reflecting continual, persistent biofilm formation. Taken together, these results indicate that ∆*ctaA* cells form a biofilm faster than WT cells, but that the biofilm formed is weaker than that formed by WT cells. This weakness could be linked to the inability of ∆*ctaA* cells to autoaggregate [44].

#### 2.3.5. Sporulation

Like biofilm formation, sporulation is a strategy used by *B. cereus* to adapt to and survive a variety of stresses [45,46]. We found no difference in the ability of WT and ∆*ctaA* strains to sporulate (data not shown), in contrast to reports for *B. subtilis* [3].

## 3. Discussion

The aim of this study was to investigate how mutations in the *ctaA* gene-identified during a proteomics screen of bacteria surviving in groundwater-affected the capacity of *B. cereus* AH187 to grow and resist exogenous stress.

Pathogenic variants often emerge due to increased fitness, which can result from point mutations [47,48]. *CtaA* is a target gene for spontaneous mutation in both *B. cereus* [2] and *B. subtilis* [3]. This gene encodes an integral transmembrane protein displaying heme O monooxygenase activity. The results presented here show that *ctaA* gene deletion inactivates cytochrome *caa3* oxidase in *B. cereus* AH187 (Figure 2b). Consequently, we can conclude that CtaA plays a role in cytochrome *caa3* biogenesis in *B. cereus* AH187, and that cytochrome *caa3* activity requires heme A. Like *caa3*, cytochrome *aa3* quinol oxidase binds heme A. However, in the absence of heme A, the active *bo3* variant, which binds heme B and heme O instead of heme A can replace cytochrome *aa3* oxidase [8]. Our proteomics data indicate that cytochrome *aa3* apoprotein, encoded by QoxABCD, is over-produced in *ctaA* mutants. We hypothesize that disruption of the *bc*-*caa3* branch of the respiratory network could redirect electron flow toward menaquinol-cytochrome *bo3* oxidase. This redirection probably generates redox stress, as reflected by increased levels of key proteins involved in the redox stress-response. These proteins include the flavohemoglobin Hmp, which may promote cytochrome *bo3* activity [49]. Redox stress is associated with reorganization of the respiratory network, and could result from excess ROS production due to blocked electron flow within the *bc* complex leading to accumulation of reduced quinone [50,51]. However, it is also possible that loss of CtaA disrupts plasma membrane integrity, leading to secondary redox stress [52].

*B. cereus* AH187 ∆*ctaA* exhibited a small-colony phenotype on LB solid media (Figure 2a). A similar phenotype was observed in *B. subtilis* and *S. aureus ctaA* mutants [3,53], and has been demonstrated to allow bacteria to survive in hostile environments, by reducing metabolic needs [54]. When grown in liquid medium supplemented with glucose, *B. cereus* AH187 ∆*ctaA* displayed no significant growth defect, in line with what was reported for the spontaneous *ctaA* mutant of *B. cereus* strain 9373 [55]. This capacity to grow in suspension is probably linked to the ability of bacteria to increase acetate overflow to overcome respiration dysfunction. Indeed, increased acetate overflow could prevent NADH/NAD^+^ imbalance in the respiratory chain and allow faster ATP production, through acetate kinase activity, to meet the requirements for biomass [56,57]. Overflow metabolism has been suggested to be advantageous for bacteria using this strategy to compete with fully respiration-competent cells in both nutrient-rich and -poor medium [58]. Increased overflow metabolism, due to loss of CtaA, could thus increase the overall fitness of bacteria.

Indeed, our results show that CtaA deficiency makes *B. cereus* more resistant to cold and oxidative stresses, suggesting that *ctaA* deletion generates an effective redox response to various types of stress [59].

Interestingly, loss of CtaA also changed the surface composition of *B. cereus*. For example, levels of one of the putative S-layer proteins (B7HXP4) was decreased 2-fold (Table S3). Production of the S-layer demands a high metabolic investment from micro-organisms [60], mainly due to the high synthesis rate of its proteins (means of normalized spectral abundance factor (NSAF) for B7HXP4 = 0.59 ± 0.18%, 0.50 ± 0.10% and 0.73 ± 0.13% at EE, LE and S-growth phase in WT strain, Table S1). The flagellin protein FliC is also produced in large quantities (means of NSAF = 0.37 ± 0.03%, 1.70 ± 0.45% and 1.50 ± 0.03% at EE, LE and S-growth phase in WT strain, Table S1), and was similarly found to be 2- to 4-fold less abundant in Δ*ctaA* compared to the WT strain (Table S3). Thus, *ctaA* deletion affected *B. cereus* motility, as reported in *S. aureus* [61]. By decreasing the synthesis of S-layer protein and flagellin, bacteria may be attempting to conserve energy that can then be diverted to mount a stress-response [62] and promote survival through other processes, such as sporulation.

Flagella and surface proteins are involved in autoaggregation of planktonic bacteria, and biofilm formation [41]. Both these processes play important roles in bacterial survival in their natural environment and in diseases [63]. Our results indicate that the Δ*ctaA* strain lost the autoaggregation phenotype but retained its ability to form biofilm on a solid surface. However, the biofilm formed by Δ*ctaA* cells appeared weaker than that formed by WT cells, probably due to the loss of the autoaggregation phenotype. Lack of autoaggregation within a biofilm could be a survival advantage for bacterial cells in limited nutrient conditions [41].

In conclusion, CtaA synthesis is dispensable for *B. cereus* growth under oxic conditions as bacteria can implement an effective compensation strategy. This strategy is closely linked to the flexibility of the respiratory network and the bacteria’s ability to generate a respiration-dysfunction-mediated stress-response. Considering the phenotypes of Δ*ctaA* cells, we propose that spontaneous inactivation of *ctaA* could improve *B. cereus*’ fitness in limited nutrient conditions, such as those encountered in groundwater, as in the study where we initially identified mutation of this gene.

Better understanding how bacterial aerobic respiration, and terminal oxidases, participate in pathogen fitness in nutrient-limited environment is an exciting research challenge. Proteomics approaches offer great potential for characterizing respiratory chain in model strains. However, further studies utilizing multi-omics approaches are necessary to address the mechanisms by which bacteria regulate their metabolism in response to respiration dysfunction.

## 4. Materials and Methods

### 4.1. Bacterial Strain and Mutant Construction

The wild-type Bacillus cereus AH187 (F4810/72) strain used in this study originates from emetic food poisoning outbreak [64]. Deletion of the ctaA (BCAH187_A4064) gene was achieved by allelic replacement, using the temperature-sensitive pMAD plasmid [65]. Briefly, 1-kbp flanking DNA sequences upstream and downstream of the BCAH187_A4064 gene were amplified using the appropriate oligonucleotide primers (Table S5). The recombinant PCR products containing DNA sequences upstream and downstream of the BCAH187_A4064 gene were cloned into the pCR-TOPO 2.1 plasmid (TOPO T/A cloning kit, Invitrogen). The resulting plasmid, pctaA-KO, was digested with SmaI (Promega, Charbonnières-les-Bains, France) and ligated with a SmaI-digested DNA fragment encoding spectinomycin resistance. The new plasmid, pctaA-KO-spec, was digested with EcoRI (Promega, Charbonnières-les-Bains, France), and the resulting fragment was cloned into pMAD digested by the same enzyme. The recombinant plasmid, pMAD ctaA-KO-spec, was used to transform B. cereus AH187. Chromosomal allele exchange was confirmed by PCR with oligonucleotide primers located upstream and downstream of the DNA regions used for allelic exchange (ExF4064 and ExR4064, Table S5, Figure S3). For complementation assays, the plasmid pHT304-ctaA [2] was electroporated in ∆ctaA strain and then transformants were selected on LB plate containing erythromycin (Sigma Aldrich, Saint Louis, CA, USA) and confirmed by PCR.

### 4.2. Growth Parameters and Analytical Procedures

Growth of *B. cereus* WT and ∆*ctaA* strains was performed as described previously [2], and monitored spectrophotometrically at 600 nm (BioSpec-mini, Shimadzu Biotech). Growth parameters in MOD medium [14] supplemented with 30 mM glucose or 60 mM glycerol (all from Sigma Aldrich, Saint Louis, CA, USA), in the presence or absence of 5 µM antimycin A (from *Streptomyces* sp., Sigma Aldrich, Saint Louis, CA, USA) were studied on microtiter plates in a temperature-controlled, automated optical density reader (Flx-Xenius XMA, Safas, Monaco). The maximal specific growth rate (µ_max_) was calculated using the modified Gompertz equation [66]. Glucose and acetate concentrations were determined in filtered supernatants using enzymatic kits purchased from Biolabo (Maizy, France) and BioSenTec (Auzeville Tolosane, France), respectively. Kits were used according to the manufacturer’s protocols.

### 4.3. TMPD Oxidase Staining

The presence of active *c*-type cytochrome oxidase was verified by a colorimetric assay using N, N, N′, N′-tetramethyl-p-phenylenediamine (TMPD) as an artificial electron donor that can be oxidized by cytochrome *caa3* to a blue colored product that stains colonies. TMPD oxidase staining was performed by adding drops of oxidase reagent (bioMerieux, Craponne, France) to *B. cereus* colonies grown overnight at 30 °C on LB plates.

### 4.4. Sample Preparation for Shotgun Proteomics

WT and ∆ctaA strains were grown in MODG medium, as previously described [14]. For proteomics analysis, cultures (three biological replicates) were performed in 2-L flasks containing 500 mL culture medium. Flasks were incubated with shaking (200 rpm) at 30 °C. The inoculum was a sample of an overnight culture harvested by centrifugation, washed and diluted in fresh medium to obtain an initial optical density at 600 nm (OD_600_) of 0.02. Samples (100 mL) were collected at EE (OD_600_ = 0.1), LE (OD_600_ = 1) and S growth phases (OD_600_ = 1.5) (Figure S1). Protein and peptide samples from cells and culture supernatants were prepared, as previously described [2].

### 4.5. Protein Identification by LC–MS/MS and Label-Free Quantification

Peptides were separated on an Ultimate 3000 nano LC system coupled to a Q-Exactive HF mass spectrometer (Thermo Fisher Scientific, Illkirch-Graffenstaden, France) for analysis. Briefly, peptide mixtures (10 µL) were loaded, desalted online on a reverse-phase Acclaim PepMap 100 C18 precolumn (5 mm, 100 Å, 300 µm i.d. × 5 mm), and then resolved according to their hydrophobicity on a nanoscale Acclaim Pepmap 100 C18 column (3-μm bead size, 100-Å pore size, 75 µm i.d. × 500 mm) at a flow rate of 200 nL·min^−1^ using a bi-modal gradient combining buffer B (0.1% HCOOH, 80% CH_3_CN, 20% H_2_O) and buffer A (0.1% HCOOH, 100% H_2_O). Peptide digests of cellular proteins were eluted by applying a 90-min gradient (4–25% B in 75 min, followed by 25–40% B in 15 min), whereas extracellular proteins were eluted by applying a 60-min gradient (4–25% B in 50 min, followed by 25–40% B in 10 min). A Top-20 method was used, with full MS scans acquired in the Orbitrap mass analyzer over an m/z range from 350 to 1500, at 60,000 resolution. After each scan, the 20 most abundant precursor ions were sequentially selected for fragmentation and MS/MS acquisition at 15,000 resolution. A 10-s dynamic exclusion window was applied to increase the detection of low-abundance peptides. Only double- and triple-charged ions were selected for MS/MS analysis.

Sequences were assigned using the Mascot Daemon search engine (version 2.5.1, Matrix Science) against the *B. cereus* AH187 NCBI_20200622 database (7100 sequences). Peptide mass tolerance and MS/MS fragment mass tolerance were set to 5 ppm and 0.02 Da, respectively. The search included carbamidomethylation of cysteine residues (C) as fixed modification; oxidation of methionine (M) and deamidation of asparagine and glutamine (NQ) were included as variable modifications. All peptide matches with a peptide score associated with a Mascot *p*-value lower than 0.05 were retained. Proteins were considered valid when at least two distinct peptides were detected in the same sample, resulting in a false discovery rate lower than 1%. NSAF values were calculated by dividing the number of spectra assigned to a protein in a given sample by its molecular weight, as recommended [67]. Results were then statistically analyzed. The R tool Bioconductor DEP package (version 1.12.0) was used to perform PCA and determine changes in protein abundance between WT and ∆*ctaA* mutant at the different growth phases [68]. Significant changes were selected where the adjusted *p*-value was lower than 0.05 and the |fold-change| ≥ 1.5 (|log_2_ fold-change| ≥ 0.56).

The mass spectrometry proteomics data have been deposited to the ProteomeXchange Consortium via the PRIDE partner repository with the dataset identifier PXD030118 and 10.6019/PXD030118 for cellular proteome of *B. cereus* AH187, PXD030114 and 10.6019/PXD030114 for cellular proteome of ∆*ctaA* mutant, PXD030165 and 10.6019/PXD030165 for exoproteome of *B. cereus* AH187 and PXD030163 and 10.6019/PXD030163 for exoproteome of ∆*ctaA* mutant.

### 4.6. S-Layer Extraction and Western Blot Analysis

S-layer extraction was performed as described in [69]. Culture samples of WT, ∆*ctaA* and complemented ∆*ctaA*(p*ctaA*) strains harvested at EE, LE and S growth phases were centrifuged for 5 min at 8000× *g*. Pellets were washed with PBS and boiled (100 °C) for 10 min in 110 μL PBS–3 M urea to extract S-layer and S-layer-associated proteins. Extracts were then centrifuged 10 min at 16,000× *g*, and the S-layer extracts were separated from bacteria pellets. S-layer extracts were added to 1/5 volume of sample buffer (10% SDS, 1% β-mercaptoethanol, 50% glycerol, 300 mM Tris-HCl (pH 6.8), 0.1% bromophenol blue). The protein content of cell lysates was estimated by BCA assay (Pierce). S-layer extract aliquots (2 µg) were separated on 10% SDS-PAGE gels, and transferred to nitrocellulose membranes (Thermo Fisher Scientific, Illkirch-Graffenstaden, France) for immunoblotting. Proteins were detected using rabbit antiserum raised against purified B7HXP4 (Boutonnet et al., in preparation). Immunoreactive products were revealed by chemiluminescent detection after incubation with horseradish peroxidase (HRP)-conjugated anti-rabbit antibody (Sigma-Aldrich, Saint-Louis, CA, USA).

### 4.7. Cell Survival at 4 °C

Overnight cultures of WT, ∆*ctaA* and complemented ∆*ctaA*(p*ctaA*) strains were inoculated in 15 mL MODG media in 50 mL tubes at an initial OD_600_ of 0.02. Cultures were incubated with shaking (200 rpm) at 30 °C. Once cultures had reached exponential phase, the tubes were incubated at 4 °C with shaking for up to 576 h (24 days). The number of surviving CFU was determined by plating 100-µL volumes of 10-fold serial dilutions of cultures on LB agar plates. The colonies formed after incubation for 18 h at 30 °C were counted. All experiments were performed in triplicate.

### 4.8. Hydrogen Peroxide Killing Assays

WT, ∆*ctaA* and complemented ∆*ctaA*(p*ctaA*) strains were grown to mid-log phase (OD_600_~0.3) in MODG medium. Hydrogen peroxide challenge assays were performed by exposing samples to 10 mM H_2_O_2_ for 5, 10 or 20 min. Cells were then centrifuged and resuspended in an equal volume of H_2_O. Sample aliquots (100 μL) were appropriately diluted in H_2_O and plated on LB agar. CFUs were counted after overnight incubation at 30 °C. All experiments were performed at least in triplicate.

### 4.9. Autoaggregation

WT and ∆*ctaA* strains were grown overnight in Brain Heart Infusion (BHI) at 30 °C. OD_600_ values were adjusted to 1, and 1 mL of culture was placed in spectrophotometer cuvette. Optical density (OD_600_) was monitored over 8 h static incubation. Results were expressed as percentage of initial OD_600_. Experiments were performed in triplicate.

### 4.10. Motility

Tubes containing semisolid medium (10% tryptone, 2.5% yeast extract, 5% glucose, 2.5% sodium hydrogen phosphate, and 0.3% agar) were inoculated by stabbing down the center with a 3 mm loopful of culture, and incubated for 18 h at 30 °C.

### 4.11. Bacterial Adhesion (BioFilm Ring Test^®^)

The BioFilm Ring test [43] measures the immobilization of magnetic beads by attached cells. The more beads entrapped by cells, the fewer remain mobile. The size of the spot of free beads formed upon magnetization of the plate can be used to estimate the number of cells engaged in forming biofilm on the plate’s surface.

WT and ∆*ctaA* strains were grown in BHI at 25 and 30 °C for 24 h. Cultures were adjusted to 2 × 10^6^ CFU/mL, and 200 µL was distributed in 96-well polystyrene plates. No bacteria were added to control wells. Adhesion was measured after 4, 8 and 24 h incubation. The ability of each strain to adhere was assessed based on the BioFilm Index (BFI), calculated using the Biofilm Control software (Biofilm Control, Saint Bauzire, France) from the size of the black spot in the bottom of the wells detected by the Scan Plate Reader. BFI values are inversely proportional to attached cell number. ΔBFI (BFIcontrol−BFIsample) was calculated by subtracting the BFI for each sample from the mean BFI obtained for the controls–containing no bacteria–to assess the ability of strains to adhere. Four replicates (four wells) were analyzed for each strain and condition tested.

### 4.12. Statistical Analyses

Data from three biological replicates were pooled for statistical analyses. Comparisons of multiple data were analyzed by analysis of variance (ANOVA) followed by post hoc analysis (one-way ANOVA followed by Tukey’s post hoc analysis for stress response studies, two-way ANOVA followed by Bonferroni post hoc analysis for biofilm studies). Changes in autoaggregation ability and metabolite production were evaluated using Student’s t-test. Statistical analyses were performed using GraphPad Prism software version 6.0 (GraphPad Software, San Diego, CA, USA). *p*-values ≤ 0.05 were considered significant.

## Data Availability

The mass spectrometry proteomics data have been deposited to the ProteomeXchange Consortium via the PRIDE partner repository with the dataset identifier PXD030118 and 10.6019/PXD030118 for cellular proteome of *B. cereus* AH187, PXD030114 and 10.6019/PXD030114 for cellular proteome of ∆*ctaA* mutant, PXD030165 and 10.6019/PXD030165 for exoproteome of *B. cereus* AH187 and PXD030163 and 10.6019/PXD030163 for exoproteome of ∆*ctaA* mutant.

## Supplementary Materials

The following are available online at https://www.mdpi.com/article/10.3390/ijms23031033/s1.
