# Supplementary material for: Heme A Synthase Deficiency Affects the Ability of Bacillus cereus to Adapt to a Nutrient-Limited Environment
Source: Int J Mol Sci. 2022 Jan 18;23(3):1033. doi: 10.3390/ijms23031033 (PMC8835132; doi:10.3390/ijms23031033)
Supplement: Supplementary file 1 [file ijms-23-01033-s001.zip › Figure S1.pdf]

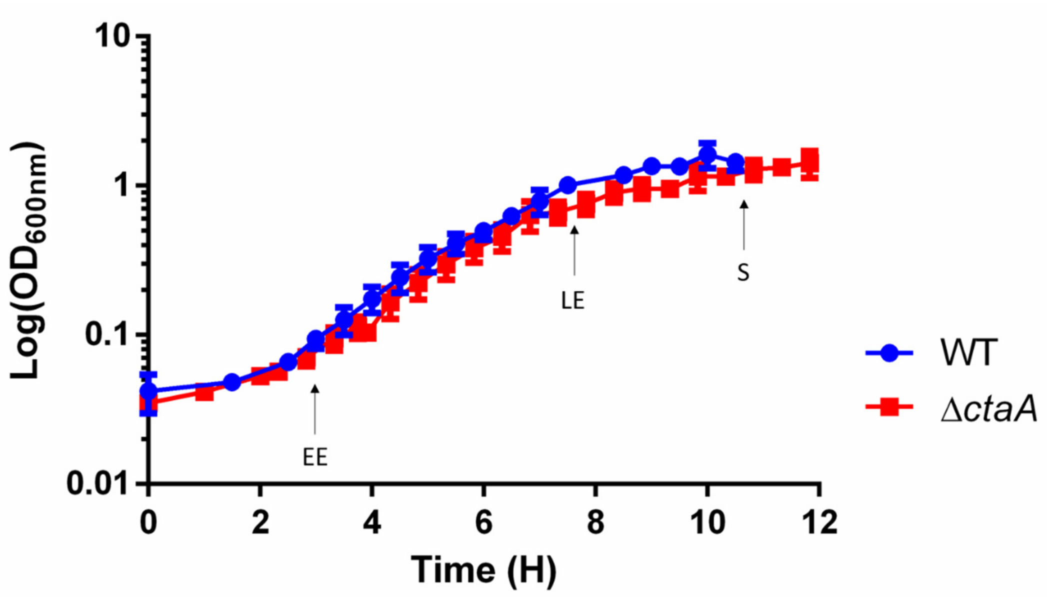

Figure S1. Growth curves of Δ*ctaA* mutant and its parental strain *B. cereus* AH187 in MODG at 30 °C. Values correspond to mean ± SD measured for three biological replicates. Samples for proteomics analyses were harvested at early exponential (EE), late exponential (LE), and stationary (S) growth phases, as indicated. The maximum growth rates (μ_{max}) were similar for WT ($0.64\pm0.02\ h^{-1}$) and Δ*ctaA* strains ($0.61\pm0.07\ h^{-1}$). Final biomasses (OD_{600nm}) were 1.61±0.31 for WT and 1.48±0.19 for Δ*ctaA*. Arrows indicate the sampling times for proteomics analysis.
