# Supplementary material for: Heme A Synthase Deficiency Affects the Ability of Bacillus cereus to Adapt to a Nutrient-Limited Environment
Source: Int J Mol Sci. 2022 Jan 18;23(3):1033. doi: 10.3390/ijms23031033 (PMC8835132; doi:10.3390/ijms23031033)
Supplement: Supplementary file 1 [file ijms-23-01033-s001.zip › Figure S2.pdf]

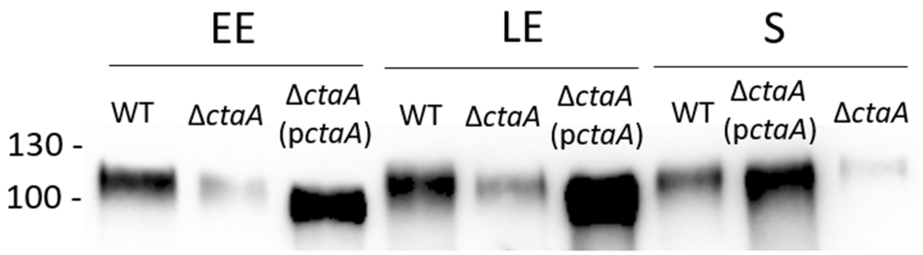

Figure S2. B7HXP4 abundance in S-layer extracts from WT, Δ*ctaA* and complemented Δ*ctaA*(p*ctaA*) strains. Proteins extracted at early exponential (EE), late exponential (LE), and stationary (S) growth phases were separated on SDS-PAGE and transferred onto nitrocellulose membranes for immunoblotting with polyclonal anti- B7HXP4 serum.
