# Supplementary material for: Heme A Synthase Deficiency Affects the Ability of Bacillus cereus to Adapt to a Nutrient-Limited Environment
Source: Int J Mol Sci. 2022 Jan 18;23(3):1033. doi: 10.3390/ijms23031033 (PMC8835132; doi:10.3390/ijms23031033)
Supplement: Supplementary file 1 [file ijms-23-01033-s001.zip › Figure S3.pdf]

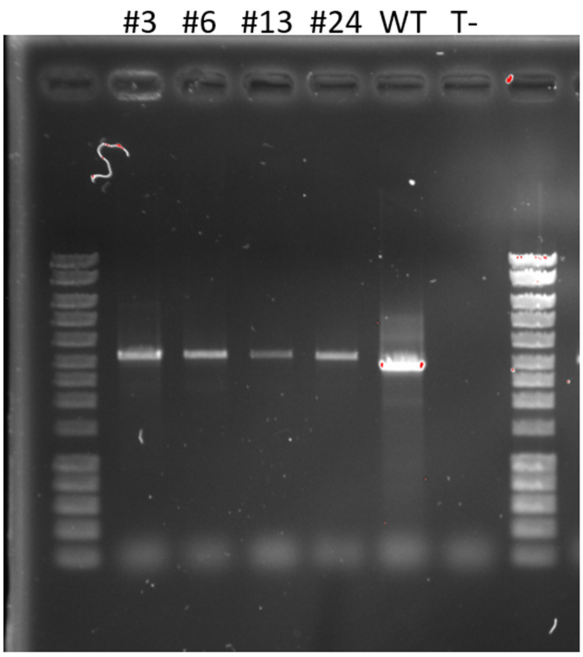

Figure S3. PCR result of *ctaA* deletion. PCR was performed on 4 Δ*ctaA* candidates (3, 6, 13 and 24) and WT strain with oligonucleotide primers located upstream and downstream of the DNA regions used for allelic exchange (ExF4064 and ExR4064, Table S5) giving 3.5kb fragment for *ctaA* deletion and 3kb for the WT.
