# Supplementary material for: Heme A Synthase Deficiency Affects the Ability of Bacillus cereus to Adapt to a Nutrient-Limited Environment
Source: Int J Mol Sci. 2022 Jan 18;23(3):1033. doi: 10.3390/ijms23031033 (PMC8835132; doi:10.3390/ijms23031033)
Supplement: Supplementary file 1 [file ijms-23-01033-s001.zip › Table S5.pdf]

Table S5. *B. cereus* strains, plasmids, and oligonucleotides used in this study

Strain, plasmid or oligonucleotide	Relevant genotype or sequence (5′→3′)	Source or reference
**Strains**		
AH187 (F4810/72)	Emetic *Bacillus cereus* reference strain	[64]
Δ*ctaA*	F4810/27 Δ*ctaA, spec*	This study
Δ*ctaA* (p*ctaA*)	F4810/27 Δ*ctaA, spec* complemented with p*ctaA*	This study
**Plasmids**		
pCR2.1 topo	Cloning vector	Invitrogen
p*ctaA*-KO	pCR2.1 derivative harboring 1000 pb upstream and downstream *ctaA*	This study
p*ctaA*-KO-*spec*	p*ctaA*-KO derivative harboring a spectinomycin resistance gene between upstream and downstream *ctaA* fragment.	This study
pMAD	Suicide vector in Gram +	[65]
pMAD *ctaA*-KO-*spec*	pMAD derivative harboring 1000 pb upstream and downstream *ctaA* interrupted by a spectinomycin resistance gene	This study
pHT304-*ctaA*	Plasmid allowing CtaA expression under its own promoter	[2]
**Oligonucleotides**		
UpF4064-bis	CATTAAGACTGCCATTGGTGTTG	
upR4064-smaI	CGGGCAAGGTATTTTCATCCCGGGGCGTTGCAATCTCTTTCACCATC	
downF4064-smaI	GTGAAAGAGATTGCAACGCCCCGGGATGAAAATACCTTGCCCGAATTGG	
downR4064-bis	GAGCGTGGACATTCTATGGACTTC	
ExF4064	CGATTGCTGCCCATCCAATTAAAGG	
ExR4064	GCACGAAATGCCAGGCGGACAGTATAG	
